# Adaptation of radiation field analyser (RFA) as optical CT scanner for gel dosimetry

**DOI:** 10.4103/0971-6203.25666

**Published:** 2006

**Authors:** S. Brindha, Vinoth Kumar, S. Vasanth, B. Ravindran Paul

**Affiliations:** Department of Radiotherapy, Christian Medical College, Vellore, Tamilnadu, India; * Department of Physics, Anna University, Chennai, Tamilnadu, India

**Keywords:** RFA, Gel dosimetry, CT scanner

## Abstract

Optical scanning is one of the emerging evaluation tools used for obtaining dose distributions in gel dosimetry. A radiation field analyzer adapted into an optical CT scanner to evaluate an irradiated Fricke gel has been already reported by others. This prototype optical CT scanner functions like a first generation x-ray CT scanner in the translate-rotate fashion. A similar scanner was constructed in our department for optical scanning of irradiated FX gel. At first, an aquarium was constructed and fitted into the water phantom of the RFA with provision to place the gel phantom to be scanned along with a light source and detector. The movements of the RFA were utilized to scan the gel phantom. A scan of a cuvette filled with colored solution was carried out and the resulting images were reconstructed and profiles obtained to evaluate the working of the optical scanner. A scan of the gel phantom was then obtained to evaluate the performance of the scanner. Thus a radiation field analyzer (DYNASCAN) was successfully adapted to an optical scanner to evaluate Fricke gels in our department.

## Introduction

Since the development of Fricke ferrous sulphate gel dosimeters in 1984 to visualize 3D dose distributions, studies have been undertaken to investigate the feasibility of using ferrous gel as a three-dimensional dosimetry system in radiation oncology.[1–5] To date, MRI is used to evaluate the radiation-induced changes in relaxation times of gel dosimeters. Even though MRI has proved to be a valuable tool for evaluation of gel dosimeters, the method contains a number of problems such as the availability of the MRI in clinical situations and artifacts caused by imperfect RF pulses, eddy currents, temperature gradient during scanning and magnetic field inhomogeneties leading to high uncertainties in the gel read-out. Although, work has been carried out to eliminate certain artifacts,[6–12] other evaluation techniques such as optical tomography,[13–14] vibrational spectroscopy,[15] X-ray computerized tomography (CT)[16] and ultrasound[17] have also been investigated.

Optical Computerized Tomography has shown a great potential for evaluating gel dosimeters. In conventional ferrous sulphate dosimetry, UV spectrophotometry is used to obtain quantitative measurement of the absorbed dose. Such changes in the optical absorption characteristics of the conventional Fricke dosimeter can be moved into the visible region of the light spectrum by a change in color brought about by the addition of xylenol orange, an ion indicator dye.[18] A new system, which is essentially a variant of the ferrous sulphate gel system by the addition of the xylenol orange ion indicator, is described.

Fricke gels are highly transparent and attenuate light mainly by absorption.[19] A laser-scanning device and a CCD imaging system have been developed for evaluating the change in optical density for ferrous sulphate, xylenol orange dye and agarose[20] for application in radiation field mapping. Since then, several investigators have developed techniques to evaluate 3D imaging of Fricke gels by tomographic reconstruction.[4,14,21,22]

Kelly *et al.*[14] proposed optical-CT scanning of FX gel with an approach analogous to first-generation X-ray CT with the X-ray source replaced by a visible laser and the X-ray detector replaced with a light-sensitive photodiode. The translate-rotate movement of the first generation CT is used in the optical CT; however, in this the object rotates and the source and the detector moves. Commercial optical scanners are not very common and developing one in a clinic is certainly a difficult task. A novel method of adapting a Radiation Field Analyzer as an optical CT scanner has already been reported.[23] In this paper, we describe the method used to convert RFA into an optical CT scanner in our department.

## Materials and Methods

The various parts of the optical scanner are i) RFA, ii) aquarium, iii) gel container, iv) detector v) light source vi) bracket and vii) data acquisition system.

### Construction of the optical CT scanner Radiation Field Analyzer (RFA)

The RFA used was a ‘Dynatech’, product of Computerized Medical System (CMS), USA, model number 3110. This consists of a Perspex water tank of dimensions 40 × 40 × 40 cm,, center of which is taken as the origin for major axes x, y and z and measuring about 300 mm in length.

A moving rail connected to a stepper motor carries the ionization chamber for radiation field analysis. The step size of the rail can be varied from 1 mm to 300 mm and the limit of movement can also be fixed to our desired value. The rail can be moved diagonally as well as in any direction along the three axes. The movement of the rail and the output from the ion chamber are controlled and measured by a computer. The number and size of the grid points or measurement points, as well as the normalization points and the origin, are user specific.

‘Dynatest’ is an option provided in this scanner to test the operational condition of the water phantom, which in turn has another option of ‘test water phantom’. With this option, the probe used for measurement could be moved in any one of the three axes at any depth fifteen times continuously. The time taken for this is 37 s. This option was utilized to adapt the RFA into an optical CT scanner for reading the irradiated gel.

### Aquarium

**Figure 1:**
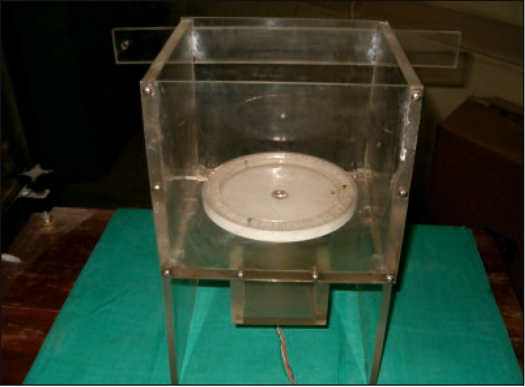
The aquarium for placing the gel phantom attached to a rotating table graduated in degrees

An aquarium made up of Perspex of dimensions 20 × 23 × 20 cm with a rotating turntable [Figure 1] was designed to mount the gel container within the RFA. This table could be rotated in steps of 1 degree using a computer-controlled stepper motor. The aquarium was filled with water to nearly match the refractive indices of the walls of the gel container to minimize the effect of deflection of laser from its path as it moved in different mediums.

### Gel container

**Figure 2:**
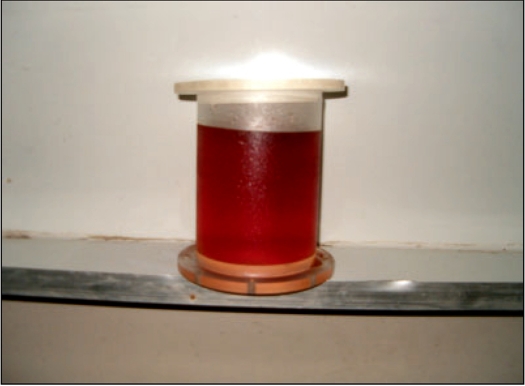
The plastic drink bottle modified as a gel container filled with unirradiated gel

The middle part of a 2-liter plastic beverage bottle was used as the gel container. The dimensions of the container were 16 cm length, 10 cm diameter and a wall thickness of 0.1 cm. This bottle was fixed on a circular Perspex plate of thickness 1.5 cm and closed with a second Perspex circular lid to maintain the cylindrical shape of the container. Figure 2 shows one such container filled with unirradiated gel.

### Detector (LDR)

A locally available Light Dependent Resistor (LDR) was used as the detector (model No: EIP 256). The diameter of the LDR was 2 cm and the sensitive part of area 2 × 1 cm^2^ was connected with a resistor to form a voltage divider. The resistance of the detector is inversely proportional to the intensity of light falling on it. The resistance decreases as the intensity of light increases and the output voltage from the voltage divider changes accordingly. Higher the intensity, lower is the output voltage and vice versa. The output voltage (V-_out_) is given by,

Vout=V*RxR+Rx

where V is the applied voltage, R_X_ is the resistance of LDR and R is the fixed resistor (10 kW).

### Laser

A laser beam was selected as a light source for the optical scanner since it is coherent, monochromatic, does not diverge and is intense. A red diode laser pointer was used as the light source in this optical CT. The diameter of the laser beam was 2 mm. The sensitivity of detector is inversely proportional to laser intensity up to a certain limit. The intensity level of laser beam was adjusted to an optimum level for reconstruction by connecting a variable resistor in series with the laser source.

### Bracket

**Figure 3:**
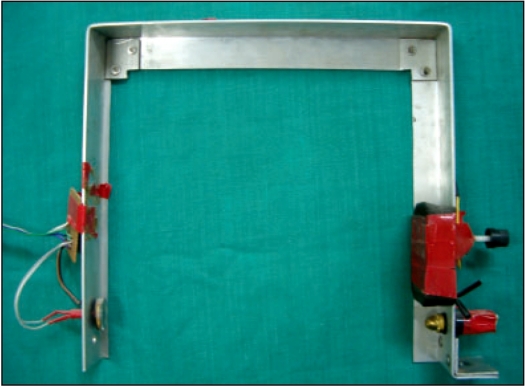
Aluminum bracket fitted with the light source and the detector

Bracket shown in Figure 3 is the most important part of the scanner since it holds the laser and the LDR detector. An aluminum plate of total length 75 cm, breadth 3.75 cm and thickness 0.2 cm was bent like a square bracket. The laser was fixed at one end of the bracket and a LDR detector was fixed exactly at the other end of the bracket. A distance of 25 cm separated the laser and detector. The bracket was fixed to the moving rail of the RFA.

### Data acquisition system

The rotation of the table in which the gel phantom was mounted was controlled by a separate computer system. The output data from the detector was also collected by the same system through 8-channel, 12-bit AD card (dynalog PCL-207). The CMS system operation and Data acquisition system operation were synchronized through software program loaded in Data acquisition system. The software program tracked the movement of rail and also collected the output data from the detector. For tracking the movement of rail, the program read the time taken between steps of rail. Thereby the two computer systems were synchronized.

The scanning speed of the moving rail of the RFA could be varied from 10 msec to 1 sec by trial and error method; a speed of 100 msec was chosen for scanning, since this was the optimum speed for the synchronization of two computer systems. The total scanning time depended upon speed of the rail.

### Working of optical CT scanner

This prototype optical CT scanner functions like a first generation X-ray CT scanner in the translate-rotate fashion. From the option of moving the probe in a single major axis, movement along y-axis was selected. A minimum step size of 2 mm was chosen for the probe movement. The area of interest for scanning was 100 mm. Since collecting data from the detector over the whole range of 300 mm of probe motion would result in a lot of unwanted data, it was decided to acquire data only through the aquarium. To achieve this, a program was developed to start the data acquisition when the output from the detector was >2 volts. This enabled to reduce the unwanted data obtained on scanning for full length of probe motion.

The data collection continued until the probe crossed the aquarium. The timer of the data collection was set according to the time taken by the probe to cross the aquarium. Once the probe crossed the aquarium and reached the other end, the phantom table rotated by 2° and the scanning continued till the probe reached the starting point. This continued for a total rotation of 180°, resulting in 90 scans. The data was transferred to a PC and then the images were reconstructed using a code written in MATLAB.

### Preliminary scanning with blank solution

The working of the scanner, data collection and reconstruction procedure was first tested by scanning a cuvette filled with color solution. A cylindrical container similar to the one to be filled with gel was initially filled with a color solution, prepared by adding arbitrary amount of ink to water to make the solution reasonably dark. A cuvette was filled with relatively higher concentration of ink and placed at the center of the cylindrical container.

The RFA was initialized, which caused the moving rail to come to the origin. The aquarium was placed at the center of the RFA. The position of the aquarium was adjusted such that the laser beam passed through the center of the rotating table. The position of the laser in z-axis was fixed to the level at which the ink solution was to be scanned. The bracket was moved to a distance of -150 mm to the left of the origin. The cylindrical container filled with color solution with the cuvette at the center was fixed to the rotating table in the aquarium. Scanning was carried out in a dark room. Each scan data was collected in separate sequence of files and transferred to a personal computer for reconstruction using MATLAB. Figures 4A and 4B show the reconstructed image of the cuvette with ink and the profile through the cuvette respectively.

**Figure 4a:**
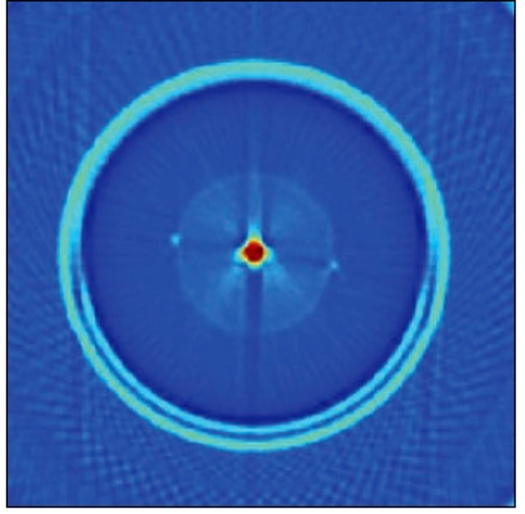
Reconstructed image of the cuvette with color solution placed within the gel container which in turn is placed inside the aquarium

**Figure 4b:**
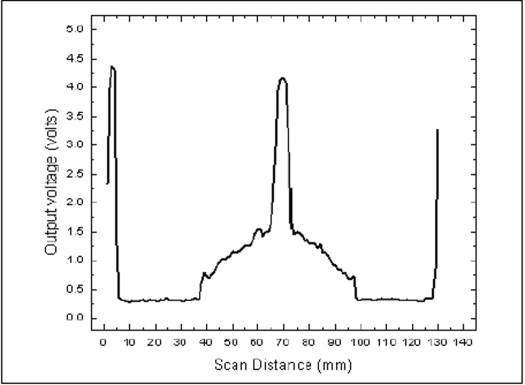
Profile taken across the reconstructed image of the cuvette filled with ink solution

### Gel preparation

The chemicals used - ferrous ammonium sulphate, xylenol orange (Sigma-Aldrich) and sulphuric acid - were of analytical reagent grade and were used without further purification. The gelling agent used in this study was analytical grade 300 Bloom Type A gelatin obtained from porcine skin supplied by Sigma-Aldrich. The gel was prepared using triple distilled water.

The FGX gel was prepared as per the recipe obtained from Queensland University of Technology, Brisbane. The components used were 5% gelatin, 0.1 mM xylenol orange, 0.1 mM ferrous ammonium sulphate and 50 mM sulphuric acid. The gel was prepared by mixing 5% gelatin with distilled water. The mixture was set to soak for 30 min and then heated up to 65°C with continuous stirring. Once the temperature reached 65°C, the mixture was removed from heat and was allowed to cool to 40°C. At 40°C, 0.1 mM xylenol orange, 0.1 mM ferrous ammonium sulphate and 50 mM sulphuric acid were added and stirred well. The gel solution was poured into a cylindrical container (plastic bevarage bottle) of dimensions 16 cm length and 10 cm diameter and was left in the refrigerator for setting.

### Pre-irradiation scanning

**Figure 5:**
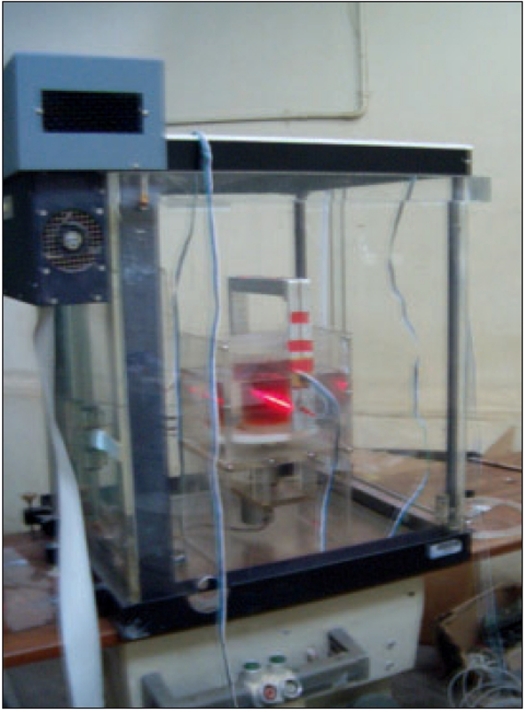
The gel container with gel placed inside the aquarium ready for scanning

The freshly prepared FXG gel was scanned before it was irradiated. The gel container was placed within the aquarium, which in turn was fixed to the rotating table as shown in Figure 5. Pre-irradiation scanning of the gel phantom was carried out at a depth of 6 cm from the surface and the resulting data was reconstructed to obtain the change in optical attenuation within the gel after irradiation.

### Irradiation of gel

After completion of pre-irradiation scanning, the gel phantom was irradiated to a dose of 5Gy to d_max_ with a field of 5 × 5 cm^2^ at 100 cm SSD using a 6 MV photon beam from a Siemens Primus Linear Accelerator.

The irradiated gel was scanned after 30 min to enable the completion of oxidation reaction of ferrous ions to ferric ions. Scanning was then repeated at a depth of 6 cm, same as for pre-irradiation scan, for a total 180°. The data was then transferred to a PC for reconstruction.

### Reconstruction

**Figure 6:**
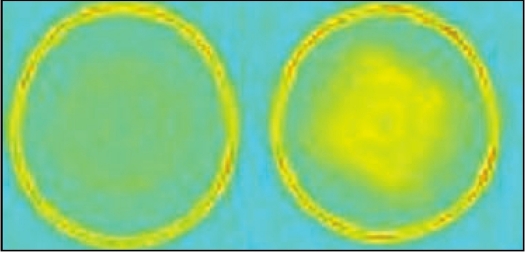
Reconstructed images of the pre-irradiated and post-irradiated gel phantom

**Figure 7:**
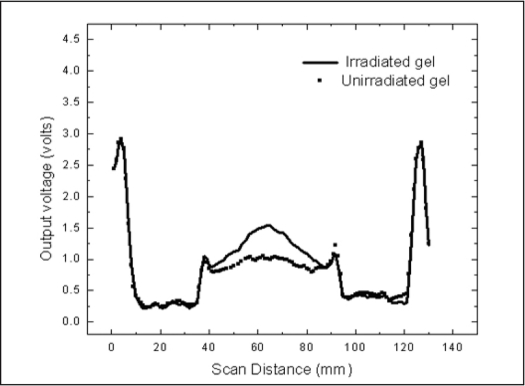
Combined profiles of the pre- and post-irradiated scans

After acquisition, the projection data were transferred to an image reconstruction program coded in MATLAB. The logarithm of the ratio of the post- and pre-irradiation projection data was calculated and the program reconstructed a two-dimensional image of the distribution of optical density with the IRADON function implemented in Matlab that uses the method of filtered back-projection. Figure 6 shows the reconstructed images of the unirradiated and irradiated gel and Figure 7 shows the combined profiles through the reconstructed images of the unirradiated and irradiated gel phantom.

In the reconstructed image [Figure 4A] A of the cuvette filled with color solution, the area of the cuvette is clearly visualized as a bright square spot. This bright part is seen as a sharp peak in the profile [Figure 4B]. Apart from the central peak, two additional peaks, numbered 1 and 2, are also seen in the profile. These peaks denote the wall of the aquarium within which the gel container with cuvette is placed.

Figure 6 shows the reconstructed images of the unirradiated and irradiated gels. The reconstructed image of the irradiated gel shows a distinct hypo dense region in the area of irradiation when compared to that of the unirradiated image. The profiles obtained from the reconstructed images of both the unirradiated and irradiated gel are compared in Figure 7. Similar to the profile shown in Figure 5b, these profiles also show two distinct peaks, numbered 1 and 2, representing the wall of the aquarium. These two peaks are due to the total attenuation of the laser beam in the walls of the aquarium. These were reproduced as the first large circle in the reconstructed image shown in [Figure 6].

The peaks numbered as 2 in Figure 7 were those derived at gel container walls. These were due to small deflections of laser at the walls. This formed the inner small circle in the reconstructed image of the gel phantom and this also helps in identifying the positioning of the gel phantom.

The profiles shown in Figure 7 are those of the unirradiated and irradiated gel phantoms. The irradiated region in the gel is seen as a smooth peak in the profile, whereas no such peak is seen for the unirradiated gel. The perfect overlapping of the peaks of both the aquarium wall and the gel phantom wall shows the perfect repositioning of the gel phantom after irradiation. This also acts as a proof for reproducibility in the rotational movement of the gel aquarium as well the moving rail of the RFA.

## Conclusions

The RFA was successfully adapted as optical CT scanner with an aquarium and a bracket fixed with a light source and a detector. The adapted scanner could be reverted to RFA for beam data acquisition after removing the aquarium and the bracket. The aquarium can be varied up to a maximum size that is convenient to fixing it into the RFA. This will enable to increase the gel phantom size, thereby making it possible to be used as a verification tool for 3D conformal radiotherapy and intensity-modulated radiotherapy. Thus any RFA with appropriate facility for the probe movement in one axis for a number of times can be adapted as an optical CT scanner with an aquarium and bracket to hold the laser light and the detector.
